# Circulating Tumor DNA Detection by Digital-Droplet PCR in Pancreatic Ductal Adenocarcinoma: A Systematic Review

**DOI:** 10.3390/cancers13050994

**Published:** 2021-02-27

**Authors:** Marisol Huerta, Susana Roselló, Luis Sabater, Ana Ferrer, Noelia Tarazona, Desamparados Roda, Valentina Gambardella, Clara Alfaro-Cervelló, Marina Garcés-Albir, Andrés Cervantes, Maider Ibarrola-Villava

**Affiliations:** 1 Department of Medical Oncology, INCLIVA Biomedical Research Institute, University of Valencia, 46010 Valencia, Spain; mhuerta@incliva.es (M.H.); srosello@incliva.es (S.R.); aferrermartinez@incliva.es (A.F.); noetalla@incliva.es (N.T.); droda@incliva.es (D.R.); vgambardella@incliva.es (V.G.); andres.cervantes@uv.es (A.C.); 2 CIBERONC, Medical Oncology Unit, INCLIVA Biomedical Research Institute, University of Valencia, 46010 Valencia, Spain; 3 Liver, Biliary and Pancreatic Unit, Department of Surgery, Hospital Clínico Universitario of Valencia, University of Valencia, INCLIVA Biomedical Research Institute, 46010 Valencia, Spain; luis.sabater@uv.es (L.S.); marina.garces@uv.es (M.G.-A.); 4 Department of Pathology, INCLIVA Biomedical Research Institute, University of Valencia, 46010 Valencia, Spain; clara.alfaro@uv.es

**Keywords:** ctDNA, digital-droplet PCR (ddPCR), pancreatic cancer, pancreatic ductal adenocarcinoma (PDAC)

## Abstract

**Simple Summary:**

Pancreatic cancer is a digestive tumor that is most difficult to treat and carries one of the worst prognoses. The anatomical location of the pancreas makes it very difficult to obtain enough tumor material to establish a molecular diagnosis, so knowing the biology of this tumor and implementing new targeted-therapies is still a pending issue. The use of liquid biopsy, a blood sample test to detect circulating-tumor DNA fragments (ctDNA), is key to overcoming this difficulty and improving the evolution of this tumor. Liquid biopsies are equally representative of the tissue from which they come and allow relevant molecular and diagnostic information to be obtained in a faster and less invasive way. One challenge related to ctDNA is the lack of consistency in the study design. Moreover, ctDNA accounts for only a small percentage of the total cell-free circulating DNA and prior knowledge about particular mutations is usually required. Thus, our aim was to understand the current role and future perspectives of ctDNA in pancreatic cancer using digital-droplet PCR technology.

**Abstract:**

Pancreatic cancer (PC) is one of the most devastating malignant tumors, being the seventh leading cause of cancer-related death worldwide. Researchers and clinicians are endeavoring to develop strategies for the early detection of the disease and the improvement of treatment results. Adequate biopsy is still challenging because of the pancreas’s poor anatomic location. Recently, circulating tumor DNA (ctDNA) could be identified as a liquid biopsy tool with huge potential as a non-invasive biomarker in early diagnosis, prognosis and management of PC. ctDNA is released from apoptotic and necrotic cancer cells, as well as from living tumor cells and even circulating tumor cells, and it can reveal genetic and epigenetic alterations with tumor-specific and individual mutation and methylation profiles. However, ctDNA sensibility remains a limitation and the accuracy of ctDNA as a biomarker for PC is relatively low and cannot be currently used as a screening or diagnostic tool. Increasing evidence suggests that ctDNA is an interesting biomarker for predictive or prognosis studies, evaluating minimal residual disease, longitudinal follow-up and treatment management. Promising results have been published and therefore the objective of our review is to understand the current role and the future perspectives of ctDNA in PC.

## 1. Introduction

There is a great need to unveil the genetic landscape of pancreatic ductal adenocarcinoma (PDAC), one of the most aggressive human malignancies. This tumor type, although not very frequent, has an increasing incidence and is the seventh leading cause of cancer-related death in both men and women worldwide [1]. Indeed, it is predicted to become the second by 2030 [2]. Globally, 495,773 new cases and 466,003 deaths of pancreatic cancer were reported in 2020 (GLOBOCAN, https://gco.iarc.fr/ (accessed date 4 February 2021)). The overall 5-year survival rate is 9%, and the median survival is still shorter than 6 months, the lowest among all cancer types [3]. This poor survival is mostly attributed to the absence of effective screening methods, late diagnosis due to non-specific symptoms, lack of sensitive or specific biomarkers for early diagnosis, propensity for early metastatic spread, and the limited therapeutic advancements over the last years [4,5]. Consequently, the prognosis of pancreatic cancer remains unimproved. The onset of symptoms and PDAC diagnosis is usually late, at an advanced stage, where most patients have metastatic disease.

**Figure 1:**
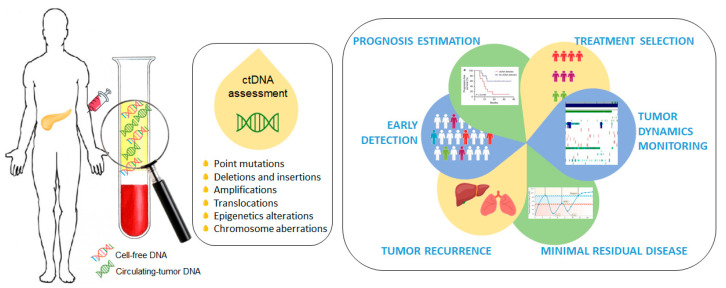
Circulating-tumor DNA (ctDNA) as a promising non-invasive blood-based biomarker in cancer management. Quantitative detection of ctDNA is based on the identification of various tumor-specific genetic or epigenetic aberrations in plasma cell-free DNA (cfDNA) samples.

Cell-free circulating tumor DNA (ctDNA) is a promising non-invasive blood-based biomarker in cancer management, and an alternative to traditional blood-based protein biomarkers [6,7]. ctDNA has shown benefit in early detection, prognosis estimation, treatment selection, tumor dynamics monitoring, minimal residual disease detection and tumor recurrence during follow up [8,9,10,11,12,13,14,15,16]. ctDNA is composed of short segments of nucleic acids and reflects the genetic and epigenetic makeup of the tumor from which it originates, making it a desirable and highly specific biomarker. ctDNA provides a better representation of the molecular composition of a malignant disease than a single section from a surgical tumor specimen or a tissue biopsy and, therefore, its clinical application in PDAC is extremely important and interesting due to the difficulty of obtaining tumor tissues (Figure 1).

One challenge related to ctDNA biology is a lack of consistency in the study design. Thus, in this article, we review current knowledge regarding ctDNA for pancreatic cancer using digital-droplet PCR (ddPCR). The publications cited were selected from the PubMed database (http://www.ncbi.nlm.nih.gov/pubmed (accessed date 27 February 2021)). Key search terms, or aliases, used were ctDNA, cfDNA, liquid biopsy, pancreatic cancer, digital PCR and ddPCR. The eligibility criteria for articles reviewed here included original studies published beyond 2015 in patients with a clinical and histological diagnosis of pancreatic adenocarcinoma that was molecularly characterized in plasma and serum liquid biopsies by digital PCR technology. Studies published in a language other than English were excluded. This paper will focus on the potential application of ddPCR to detect ctDNA for PDAC. 

## 2. Increasing Genetic Knowledge to Improve Clinical Practice

In recent years, better understanding of the molecular pathways that direct tumor progression is leading to the development of what we know as precision medicine. Although to a lesser extent than in other tumors, there have also been advances in this area in PDAC.

PDAC exome sequencing studies suggest a 20-year window opportunity for early detection before any symptoms occur [17,18]. Early identification of both the initial and recurrence disease would therefore improve clinical outcomes. Several common gene mutations are involved in PDAC carcinogenesis. The most important frequent gene mutations include *KRAS*, *CDKN2A*, *SMAD4* and *TP53*. However, their widespread use is limited by the difficulty of obtaining tumor tissues. In this context, a diagnostic and prognostic noninvasive blood test for pancreatic cancer would be very valuable. Currently, the only non-invasive blood-based biomarker routinely used in clinical practice is carbohydrate antigen 19-9 (CA 19-9). However, because of its low sensitivity (78%) and specificity (82%), it is unsuitable for screening the general population and its diagnostic utilization to detect early-stage tumors is discouraged unless in combination with other circulating biomarkers [19,20,21]. Thus, novel biomarkers that can reliably identify initial, residual or progressive disease are urgently needed.

Surgical resection is the only radical treatment with a potential chance of cure. However, at the time of diagnosis, only a few patients (10–15%) have localized disease. Molecular tumor profiling has relied on the analysis of tumor tissues obtained from surgical resection specimens in the majority of the tumor types. Unlike many other tumor types, PDAC treatment decisions are not made according to tumor biology, and patients undergo chemotherapy and radiotherapy according to their tumor stage. Until a year ago, nine different chemotherapeutic drugs were approved by the Food and Drug Administration (FDA), and some progress had been made in the management of advanced disease by the administration of multidrug regimens such as FOLFIRINOX and gemcitabine/nab-paclitaxel therapy, without any biomarkers that guide the choice of one or another treatment [22,23,24,25,26]. Fortunately, on December 2019, the FDA approved the first targeted-treatment for pancreatic cancer: olaparib. The randomized phase III POLO trial demonstrated statistically significant benefit in progression free survival (PFS), as maintenance therapy versus placebo, in patients with germline *BRCA*-mutated (gBRCAm) metastatic pancreatic adenocarcinoma whose disease has not progressed after a first-line platinum-based chemotherapy regimen [27]. Although the results are very promising, the prevalence of gBRCAm in PDAC is only 4–7% [27]. Despite these significant advances, treatment is always with a palliative intent and unfortunately, there are no long-term survivors. Thus, medical management of pancreatic cancer remains a challenge and developing new immunotherapies, and stroma-directed and targeted-therapies is an unmet need.

In 1983, Shapiro et al. first reported the presence of circulating cell-free DNA (cfDNA) in pancreatic cancer [28]. Since then, a research focus on genetic alterations in ctDNA has become mainstream.

Personalized and precision treatments are the novel goal for pancreatic cancer and involve understanding each patient’s driver genes. The analysis of ctDNA represents a unique method to explore the genetic mutation and may be translated into the clinical setting. *KRAS* represents an important potential biomarker for PDAC. It has been the best-characterized PDAC tumor-related gene due to the following reasons: (i) among human malignancies, PDAC has the highest frequency of *KRAS* mutations (up to 90%), (ii) the most frequent *KRAS* point mutations are located in codon 12, and (iii) alterations in this gene appear to occur at an early stage of pancreatic carcinogenesis. Several studies have evaluated the role of *KRAS* in the diagnosis, prognosis and treatment of PDAC [29]. In addition, the evaluation of *KRAS* mutation testing in PDAC patients has been discussed in depth for the past 20 years [30].

In 1998, Yamada et al. demonstrated that *KRAS* mutations in plasma may be clinically useful for evaluating tumor burden and treatment efficacy for pancreatic cancer [31]. In 1999, Castells and colleagues detected, for the first time, circulating mutant *KRAS* genes in plasma from PDAC patients and reported the association between poor survival and the presence of *KRAS* mutations in PDAC patients’ plasma [32]. Since then, several research studies have reported the prognosis and predictive significance of *KRAS* ctDNA detected in metastatic and perioperative settings, as well as their therapeutic evaluation along longitudinal monitoring of the disease [33,34]. However, most studies have focused on the presence of ctDNA mutations in a broad set of genes to highlight tumor heterogeneity and demonstrate clonal evolution over the course of disease progression. Despite different genetic mutations being identified in PDAC, nearly all of them have failed to facilitate a treatment approach. Moreover, PDAC tumor biology is not completely known and new-targeted therapies cannot be implemented.

ctDNA-based assays are confronted with several challenges, such as that ctDNA accounts for only a small percentage of the total cfDNA in the peripheral blood, sometimes less than 0.01%, and that prior knowledge of particular mutations is usually required, which in pancreatic cancer is hard to obtain [35,36]. However, the analysis of ctDNA has evolved since its inception with improvements in the technologies and detection limits [8]. Third-generation sequencing techniques have rapidly advanced and have the potential to expedite extensive application of ctDNA detection for routine patient management.

Currently, high-throughput next-generation sequencing (NGS) and droplet digital PCR (ddPCR) are the most promising techniques for the detection of liquid biopsy mutations. NGS-based methods generate extensive information as they allow the simultaneous evaluation and detection of multiple genetic and epigenetic aberrations over millions of ctDNA molecules. Thus, NGS leads to the discovery of novel mutated variants and presents high multiplexing capabilities. However, it is time consuming, has higher cost, requires powerful informatics support and cannot be readily applied to monitor patients longitudinally [37]. Conventional NGS methods allow a sensitivity higher than 2%. Moreover, whole-exome or whole-genome sequencing approaches usually generate around 30× to 100× average sequencing coverage, which leads to a low sensitivity detection on ctDNA [36]. Several different NGS-based technologies have been developed to enhance ctDNA detection sensitivity and specificity: Safe-Sequencing System (Safe-Seq) [38], Cancer Personalized Profiling by deep Sequencing (CAPP-seq) [39], Integrated digital error suppression-enhanced CAPP-seq (iDES-enhanced CAPP-seq) [40], or Base-Position Error Rate (BPER) [36,41]. Despite sensitivities of improved NGS-based approaches being similar to ddPCR, this paper will focus on the potential application of ddPCR to detect ctDNA for PDAC.

### 2.1. Digital-Droplet PCR Technology

In 1999, Vogelstein and Kinzler described the digital PCR (dPCR), a new microtiter plate-based technology for rare sequences detection [42]. This dPCR technology allows partitioning and individually testing of target sequences within separate compartments. dPCR sensitivity relies on the number of individual compartments and sequences created and analyzed, along with the false-positive rate of each assay [36].

**Figure 2:**
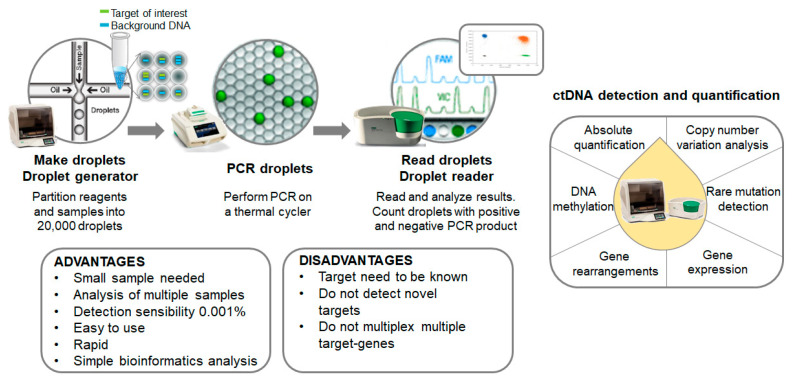
Overview of the mutation screening process. Summary of both advantages and disadvantages of the technology disclosed and other putative applications.

Digital-droplet PCR (ddPCR) is a novel next-generation technique based on nanoliter-sized water-in-oil emulsion droplet technology. At present, ddPCR is one of the most powerful methods available for the accurate quantification of a scarce amount of circulating nucleic acids in plasma. It is currently being used for absolute quantification, rare mutation detection, copy number variation analysis, DNA methylation and gene rearrangements in different types of clinical samples. Compared to NGS approaches, ddPCR experiments are easier to set up, faster, present higher sensitivity and do not require complex bioinformatics analysis (Figure 2).

BEAMing (beads, emulsion, amplification and magnetics) was the first high-throughput droplet-based digital PCR used for the detection and enumeration of genetic variants [43,44]. Despite the low limit of detection achieved, up to 0.0001%, it requires a relatively cumbersome and complicated procedure for routine clinical use [44]. Nowadays, several droplet-based digital PCR platforms are being commercialized, including Raindrop^TM^ digital PCR (Raindance Technologies, Lexington, MA, USA), Bio-Rad QX200^TM^ Droplet Digital^TM^ system (Bio-Rad Laboratories, Hercules, CA, USA) or Naica^TM^ System (Stilla Technologies, Villejuif, France) [45]. In all of these platforms, ctDNA samples are partitioned in aqueous droplets acting as independent micro-compartments. Ideally, each droplet contains one haploid genome and all the reagents needed to perform the PCR assay. Different fluorescent signals identify mutant and wild-type sequences. The most widely used is the QX200 Droplet digital PCR System (Bio-Rad Laboratories) that generates up to 20,000 nanoliter-sized droplets.

In cancer, ddPCR represents a robust method for quantifying low-abundance point mutations in cfDNA, with high sensitivity ranging from 0.001% to 0.1%, in order to reflect intratumoral heterogeneity and to track the dynamic changes in tumor burden in response to treatment during follow-up. An extensive spectrum of molecular markers has been interrogated in liquid biopsies from different tumor types using ddPCR for diagnosis, predictive and monitoring purposes. Therefore, ddPCR is a particularly promising technology due to its low turnaround time, high scalability and exquisite sensitivity [46]. Different clinical scenarios have been established, where the reliance on precision and sensitivity offered by ddPCR is the highest priority. A description of the most relevant studies of ddPCR ctDNA application in PDAC is provided in Table 1.

#### 2.1.1. ddPCR Research Approach

The high detection capability of ddPCR may permit the elucidation of alternative biomarkers for PDAC. ctDNA is found at relatively high concentrations in the circulation of most patients with metastatic disease and at lower, but detectable, concentrations in patients with localized tumors [47,62,63]. Thus, an extremely sensitive method, able to discriminate between the few mutation-associated alleles released into the bloodstream from normal variant background, is needed to follow early-stage tumor development.

Earl et al. reported plasma *KRAS* (G12D, G12V and G12R) mutant ctDNA detection using ddPCR in 8 of 31 (26%) patients across various PDAC stages. In addition, they found that *KRAS* mutant detection was significantly correlated with overall survival (OS) (HR = 12.2, *p* < 0.001) [52]. Lin et al. reported similar plasma *KRAS* results (31% ctDNA *KRAS* detection and worse overall survival association) [56]. Similarly, Hadano and collaborators used ddPCR to detect *KRAS* (G12D, G12V and G12R) mutations in plasma ctDNA from 105 PDAC patients [10]. They reported a cumulative rate of 31% ctDNA detection across stages, with a median survival of 13.6 months vs. 27.6 months in those patients with detectable vs. no detectable ctDNA, respectively, and a significant association with OS (*p* < 0.0001). They demonstrated that *KRAS* ctDNA mutations were associated with significantly poorer survival and therefore concluded that the presence of ctDNA in plasma could be an important and powerful survival predictor. Consistently, Cheng and collaborators (2020) found that both *KRAS* G12D and G12V mutations were associated with poor prognosis [51]. Kinugasa and colleagues (2015) analyzed serum *KRAS* (G12D, G12V and G12R) ctDNA mutations in 75 pancreatic cancer patients, with previously published *KRAS* mutations in a development cohort, and in 66 patients in an independent blinded validation cohort. *KRAS* ctDNA mutations were found in 62.6% and 54.5% of the development cohort and validation set samples respectively. Similarly, they found significantly shorter median survival time in patients with ctDNA *KRAS* mutations (*p* = 0.02) [55]. Serum and plasma are good tissues for detecting cancer-specific DNA and the presence of *KRAS* mutations in blood-derived DNA may be used as a prognostic biomarker for PDAC patients [47]. However, plasma has been generally used as the material for detecting ctDNA, as serum contains genomic DNA released from white blood cells during the clotting process that can interfere with ctDNA detection [64,65,66,67].

Multiplex strategies have been described, allowing one to screen for a pool of *RAS/RAF* mutations [68]. However, the subsequent identification of particular mutations is needed. Sefrioui and colleagues (2017) used ddPCR in combination with a multiplex assay to screen the seven most common *KRAS* mutations found in pancreatic cancer. This multiplex assay covers the mutation sites G12A, G12C, G12D, G12R, G12S, G12V and G13D. The authors performed ddPCR blindly from clinical data and 56% of the cases were reported to have a *KRAS* circulating mutation with a sensitivity and specificity for diagnosis of 65% and 75% respectively. Moreover, the multiplex and simplex assay results were significantly correlated [58]. Similarly, Kim and collaborators used a multiplex *KRAS* assay and demonstrated that *KRAS* mutant concentration and fractional abundance in plasma cfDNA were associated with prognosis in PDAC patients [54]. Thus, the authors suggested that cfDNA can serve as a biomarker to aid in determining who will benefit from treatment and which tumors will recur. Woo et al. (2017) also screened multiplex *KRAS* using ddPCR in a cohort of patients with locally advanced unresectable pancreatic cancer treated with chemoradiotherapy. The authors reported significantly lower concentration of cfDNA after treatment (*p* < 0.001). However, *KRAS* mutant concentration and fractional abundance was not significantly different before and after treatment. In addition, and in contrast with previous studies, overall survival and progression free survival were not related to cfDNA concentration, *KRAS* mutation concentration or fractional abundance [61].

Sugimori and collaborators (2020) detected *KRAS* mutations by ddPCR using a multiplex probe that screens 16 *KRAS* mutations (G12A, G12C, G12D, G12F, G12G, G12L, G12R, G12S, G12V and G13A, G13C, G13D, G13G, G13R, G13S and G13V). The authors reported that patients with distant metastases, except peritoneal metastases, showed a significantly higher *KRAS* mutation detection rate in serum ctDNA compared to those with locally advanced disease or peritoneal metastases. Furthermore, for those patients without a ctDNA *KRAS* mutation at the time of diagnosis, a *KRAS* mutation was detected at the time of progression of the disease. Thus, progression free survival analysis revealed that ctDNA *KRAS* mutation patients undergoing first-line chemotherapy tended to have worse progression free survival than those without a *KRAS* mutation (median 308.5 vs. 168 days, *p* = 0.07). Interestingly, they showed that, in those cases with a *KRAS* mutation in ctDNA at the time of diagnosis, the *KRAS* mutation disappeared after the initial course of chemotherapy and reappeared concurrently with or earlier than progression of the disease, highlighting the predictive factor for disease progression of PDAC patients [59].

Despite the potential of ddPCR to identify ctDNA *KRAS* mutations, most studies have analyzed *KRAS* mutations in ctDNA in order to verify the corresponding mutation in matched tumor tissues [57,69]. Finally, *KRAS* alleles have been assessed by quantitative ddPCR in a large series of patients with PDAC, pre-neoplastic pancreatic cyst and non-neoplastic pancreatic diseases [48,49]. Quantitative ddPCR found average *KRAS* MAF to be highest in baseline metastatic samples, followed by localized disease, cystic lesions and finally, non-neoplastic pancreatic diseases.

A similar approach for *KRAS* mutations has been described in other tumor types. *KRAS* is an oncogenic driver that appeared mutated in 30% of non-small cell lung carcinoma (NSCLC) and up to 50% of colon tumors. Furthermore, it is associated with a poor prognosis. The detection of *KRAS* mutations in these tumor types is a predictive biomarker for the anti-EGFR therapy. Wahl et al. reported plasma *KRAS* (G12A/C/D/S/V and G13D) mutant ctDNA detection using ddPCR in patients across various lung adenocarcinoma stages. They found that 38% of the patients had detectable *KRAS* mutation in plasma and it was significantly associated with shorter PFS and OS [70]. Similarly, Michaelidou et al. used multiplex ddPCR to quantify *KRAS* G12/G13 MAF in ctDNA from 114 pre-treated advance NSCLC patients. Again, plasma *KRAS* G12/G13 status was associated with poor patient outcome in terms of PFS and OS (*p* < 0.001, respectively) [71]. Finally, Guibert and collaborators detected *KRAS* mutations in up to 81% of the patients with a sensitivity of 78% [72]. The authors conclude that the presence of a *KRAS* mutation in cfDNA is correlated with a poor response to treatment. Thus, the detection of *KRAS* mutations in plasma could also serve as an independent biomarker of unfavorable prognosis in NSCLC patients. ddPCR is a precise and easily feasible technique for ctDNA quantification of *KRAS* mutations. However, it is important to note the limitations of this research approach. The detection of a single mutation may lead to underestimation of the true circulating tumor burden due to the stochastic nature of circulating nucleic acids. Furthermore, the majority of the studies did not evaluate hotspot mutations in codon 61, which may minimize the true sensitivity. DdPCR requires a priori knowledge of the mutant allele. Different multiplex analyses have been carried out that take into consideration probe concentrations and/or amplicon size [73,74]. Unfortunately, the actual capability is limited to 5–10 multiplex [73].

#### 2.1.2. ddPCR Validation and Monitoring Approach

Tumor mutations are not known a priori in certain liquid biopsy applications, and therefore, all tumor mutations are queried at once. Recent studies confirmed the importance in the pathogenesis of PDAC of different mutations in various genes aside from *KRAS*, such as *TP53*, *SMAD4* and *CDKN2A* [75]. NGS approaches have the potential to detect a broad range of molecular targets. Therefore, most studies have focused on the presence of ctDNA mutations in a comprehensive set of genes to highlight tumor heterogeneity and demonstrate clonal evolution over the course of disease progression. In these types of studies, ddPCR technology has been performed in order to validate NGS results and to follow-up the disease. Thus, NGS has been combined with ddPCR for liquid biopsy analysis [50,53,57,76].

Sausen et al. evaluated the utility of using somatic mutations in ctDNA to identify patients likely to recur after surgical intervention. They identified though tumor tissue whole-exome sequencing different somatic mutations likely to be detected in ctDNA of 51 patients. They used ddPCR, focusing on alterations in *KRAS*, *BRAF* and *PIK3CA*, for ctDNA detection prior and after tumor resection in localized PDAC [9]. Alterations were found in 43% of the patients at the time of diagnosis and the analyses revealed that patients with detectable ctDNA in their plasma after surgical resection were more likely to relapse (*p* = 0.02). Indeed, the authors detected ctDNA at 3.1 months after surgery, on average, compared with 9.6 months when it was clinically detectable on computed tomography scan (*p* = 0.0004). Based on these results, liquid biopsy could be a useful tool to identify residual or recurrent disease after surgical resection and early detection relapses. Similarly, Cheng and colleagues evaluated the clinical implications of ctDNA detection in metastatic pancreatic cancer patients. Firstly, they screened a panel of 60 genes in cfDNA from 10 metastatic pancreatic cancer patients through exome sequencing. Second, ddPCR was used to identify potential mutations in *BRCA2*, *EGFR*, *ERBB2*, *KDR* and *KRAS*, in a cohort of 188 metastatic pancreatic cancer patients [50]. The *KRAS* mutation rate was 72.3%, whereas *BRCA2*, *KDR*, *EGFR*, *ERBB2* exon 17 and *ERBB2* exon 27 were 11.7%, 13.8%, 13.3%, 13.3% and 6.4%, respectively. Seufferlein and coworkers analyzed the ctDNA and their corresponding tumor tissue DNA in a cohort of 20 PDAC patients by a targeted NGS and ddPCR gene panel (*KRAS*, *TP53*, *SMAD4*, *CDKN2A*, *APC*, *ATM* and *FBXW7*). The authors found mutations in up to 75% of the patients with a concordance rate of 80% between ctDNA and tumor tissue. They concluded that 96% of the mutations found in the ctDNA from naïve therapy patients were in *KRAS* and/or *TP53* [69]. Finally, Berger et al. used a seven candidate-gene NGS approach to characterize tumor tissue DNA and ctDNA samples. The mutational status observed was then validated with ddPCR when the *KRAS* and *TP53* NGS results were discordant between paired-samples. *KRAS* and *TP53* mutations in ctDNA were detected but the authors concluded that mutations in other genes, such as *SMAD4* or *ATM*, are rarely observed [77]. Pécuchet et al. reported high sensitivity and specificity for the detection of ctDNA from pancreatic or lung cancer patients using Base-Position Error Rate (BPER) NGS [41]. In a different study, Pietraz et al. evaluated the prognostic value of ctDNA detection with NGS and ddPCR in patients diagnosed with pancreatic adenocarcinoma. According to their results, the presence of ctDNA was strongly correlated with poor overall survival in patients with advanced disease (OS; 6.5 vs. 19.0 months; *p* < 0.001), and it could be an indicator of shorter disease-free survival when ctDNA is detected after surgery [13].

Watanabe et al. studied the usefulness of monitoring *KRAS* ctDNA throughout the course of the disease to predict prognosis and response to treatment in PDAC patients. In those cases, without detection of *KRAS* mutation during the first year after surgery their prognosis was better, regardless of relapse (*p* < 0.001). Furthermore, in patients receiving first-line chemotherapy in whom *KRAS* ctDNA was not detected or disappeared within 6 months, it was associated with a statistically significantly better response to treatment (*p* < 0.001) [60]. The combined strategy of NGS and ddPCR was suggested as a cost-effective and efficient method for analyzing ctDNA in PDAC patients [78].

## 3. Future Perspectives

PDAC remains a devastating disease. Extensive research efforts have focused on the discovery of early diagnostic biomarkers and efficient therapeutic approaches. Researchers and clinicians are trying to develop novel biomarkers and treatment options.

Current advances in our knowledge of the biology and clinical application of ctDNA have provided evidence that the use of ctDNA as a liquid biopsy can improve cancer diagnosis, monitoring and treatment management. Liquid biopsy is of great importance in PDAC as adequate tumor tissue is scarce. The trouble of obtaining enough tumor sample to carry out molecular studies in PDAC makes it difficult to advance the field of personalized therapy in this tumor type. For this reason, the possibility of performing genetic studies in peripheral blood takes on special relevance in PDAC and would probably stimulate the advancement in precision medicine in these patients. The advancement of liquid biopsy-based cancer research has been largely dependent on parallel advances in oncological genetics and genomics. The detection of *KRAS* mutations in plasma and serum ctDNA is one of the most frequently utilized liquid biopsy approaches for PDAC [79]. ctDNA is a valued diagnostic PDAC tool. Moreover, ctDNA is believed to play an important role in PDAC prognosis [80]. *KRAS* ctDNA MAF has been associated with PDAC clinical stage [81]. Although, a *KRAS* mutation in ctDNA is presumed to be a promising diagnostic biomarker in PDAC.

*KRAS* is one of the most common human oncogenes, being mutated in around 20–30% of all human cancers. However, *KRAS* inhibitors have largely failed and therefore, *KRAS* has earned the title of “undruggable”. Recently, the *KRAS* G12C inhibitor sotorasib has been approved by the FDA. Responses were observed in *KRAS* G12C tumor patients of different histologic subtypes, including up to 32% of NSCLC patients, 7% of colorectal patients and one pancreatic tumor patient [82]. The *KRAS* G12C mutation is the most common *KRAS* mutation in NSCLC, being around 13%, and it is between 1–3% in colorectal cancer. *KRAS* G12C mutation is rare in PDAC, being around 1% of all *KRAS* mutations. However, these results are very encouraging as a previously unapproachable target is now conceivable. The development of *KRAS* G12C targeted-agents is at an initial stage but provides hope for targeting other *KRAS* mutations as well as other undruggable targets. The detection of ctDNA *KRAS* mutations with ddPCR would make it a predictive biomarker. 

In line with this, microsatellite instability (MSI) has recently emerged as a predictive pan-tumor biomarker of immunotherapy efficacy. New innovative approaches for MSI marker detection through ctDNA using ddPCR have been developed [83]. This method is straightforward and has the potential to be routinely applied clinically in ddPCR equipped laboratories, aiding in the diagnosis and prognosis of the disease and increasing personalized treatments. 

Finally, technology is improving rapidly, and new multiplex strategies are being developed, allowing the simultaneous detection of various genetic alterations and consequently sample-saving material [84,85]. Multi-targeted ddPCR assays with gene hot spot mutations would have broad applicability for both clinical and translational research. ddPCR will become more accessible and clinically useful. However, there are still some challenges that should be addressed so that this technique may ultimately be employed into routine clinical practice.

## 4. Conclusions

Sequencing technologies develop quickly, and the understanding of ctDNA biology and clinical potential is deepening. Thus, although its application as a predictor biomarker is limited because of its low sensitivity, the eventual use of ctDNA in clinical practice seems to be assured. ddPCR has been recognized as one of the most suitable approaches for rare event detection. Furthermore, it is a cost-effective alternative to the currently used NGS platforms. However, this technology approach is only suitable for analysis when the prior knowledge about the mutation is available, emphasizing a personalized assay design. Additionally, this technology may be used in combination with NGS platforms as both methodologies can provide a robust and accurate quantitative measure of the fraction of mutant alleles.

## Figures and Tables

**Table 1:** Most relevant studies of digital-droplet PCR (ddPCR) ctDNA application in pancreatic cancer.

First author (Year) [Ref.]	Stage (N)	Sample	Method (Target)	ddPCR Results
Ako S. et al. (2017) [47]	All stages (40)	T, P, S	ddPCR (*KRAS* G12D/R/V)	- *KRAS* mutation observed in 93% DNA tissue samples and in 48% ctDNA plasma or serum samples, being G12D the most prevalent mutation - Worse prognosis in patients with plasma or serum *KRAS* mutation detected
Berger A.W. et al. (2016) [48]	Stage IV (24)	P, S	ddPCR (*KRAS* G12D/V and *GNAS* codon 201)	- ctDNA *KRAS* mutation in 41.7% of pancreas cancer patients - ctDNA *GNAS* mutation in 25% of pancreas cancer patients
Bernard V. et al. (2019) [49]	All stages (194)	T, P	ddPCR (*KRAS* G12A/C/D/R/S/V)	- >95% concordance *KRAS* mutation detection in T-P - ctDNA detection associated with shorter PFS and OS
Cheng H. et al. (2017) [50]	Stage IV (188)	P	NGS (Panel of 60 genes) ddPCR (*KRAS* G12D/R/V, *BRCA2*, *KDR*, *EGFR*, *ERBB2*)	- 72.3% of pancreas cancer patients presented with ctDNA-detected *KRAS* mutation - *KRAS* G12V and *ERBB2* exon17 mutations independent factors associated with OS - Potential clinical utility of ctDNA as a biomarker
Cheng H. et al. (2020) [51]	Stage III-IV (210)	P	ddPCR (KRAS G12D/V)	- *KRAS* ctDNA detection associated with worse OS
Del Re M. et al. (2017) [34]	Stage III-IV (27)	P	ddPCR (*KRAS* G12D/R/V, G13D)	- 70.4% patients presented ctDNA *KRAS* mutation at baseline - *KRAS* mutation changes in ctDNA associated with response to treatment
Earl J. et al. (2015) [52]	All stages (31)	P	ddPCR (*KRAS* G12D/R/V)	- *KRAS* mutation detected in all stage patients - *KRAS* mutant detection was significantly correlated with OS
Guo S. et al. (2020) [53]	Stage I-II-III (113)	P	NGS (Panel of 50 genes) ddPCR (*KRAS* G12A/C/D/R/SV)	- ctDNA *KRAS* mutation in 23.01% of the patients - *KRAS* ctDNA detection, specially G12D mutation, associated with worse OS
Hadano N. et al. (2016) [10]	All stages (105)	T, P	ddPCR (*KRAS* G12D/R/V)	- ctDNA *KRAS* mutation in 31% of the patients - ctDNA *KRAS* mutation correlated with poor PFS and OS
Kim M.K. et al. (2018) [54]	All stages (106)	P	ddPCR (*KRAS* G12A/C/D/R/S/V, G13D)	- ctDNA *KRAS* mutation was detected in 77.9% of the patients - ctDNA *KRAS* mutation correlated with worse PFS and OS
Kinugasa H. et al. (2015) [55]	All stage (75 and 66)	T, S	ddPCR (*KRAS* G12D/R/V)	- ctDNA *KRAS* mutation in 62.6% and 54.5% of the patients - Concordance rate tissue-plasma: 77.3% - Presence of *KRAS* mutation at ctDNA correlated with poor PFS and OS (not in tissue)
Lin M. et al. (2018) [56]	All stages (65)	P	ddPCR (*KRAS* G12D/R/V)	- ctDNA *KRAS* mutation in 31% of the patients - ctDNA *KRAS* detection associated with poorer OS
Mohan S. et al. (2019) [57]	Stage III-IV (55)	P	NGS (Panel of 641 genes) ddPCR (*KRAS* G12A/C/D/R/S/V, G13D)	- ctDNA *KRAS* mutation in 38.2% of the patients - ctDNA *KRAS* detection associated with poorer OS
Sausen M. et al. (2015) [9]	Stage II (51)	T, P	NGS (WES) ddPCR (*KRAS, BRAF* and *PIK3CA*)	- ctDNA *KRAS* mutation detected at diagnosis in 43% of the patients - Detectable ctDNA implies more likely to relapse and adverse prognosis
Sefrioui D. et al. (2017) [58]	All stages (56)	P	ddPCR (*KRAS* G12A/C/D/R/S/V, G13D)	- ctDNA *KRAS* mutation in 56% of the patients - ctDNA *KRAS* detection associated with tumor stage and OS
Sugimori M. et al. (2020) [59]	All stages (47)	T, S	ddPCR (*KRAS* G12A/C/D/F/G/L/R/S/V G13A/C/D/G/R/S/V)	- ctDNA *KRAS* mutation in 51.1% of the patients - ctDNA *KRAS* detection at diagnosis tended to worse PFS
Watanabe F. et al. (2019) [60]	All stages (78)	T, P	ddPCR (*KRAS* G12D/R/V, Q61H)	- -Detection of *KRAS* ctDNA during evolution was the only independent prognostic factor (not only previous to treatment)
Woo S.M. et al. (2017) [61]	Stage III (locally advanced) (44)	S	ddPCR (*KRAS* G12A/C/D/R/S/V, G13D)	- ctDNA *KRAS* mutation detection did not differ before and after treatment - ctDNA *KRAS* detection was not associated with PFS or OS

Ref.: Reference; N: Number of patients included; T: tissue; P: plasma; S: serum; WES: whole-exome sequencing; PFS: progression free survival; OS: overall survival.

## Data Availability

No new data were created or analyzed in this study. Data sharing is not applicable to this article.
